# A non-fluorescent HaloTag blocker for improved measurement and visualization of protein synthesis in living cells

**DOI:** 10.12688/f1000research.23289.2

**Published:** 2020-06-08

**Authors:** Laurie D. Cohen, Ayub Boulos, Noam E. Ziv

**Affiliations:** 1 Faculty of Medicine, Rappaport Institute and Network Biology Research Laboratories, Technion - Israel Institute of Technology, Haifa, 32000, Israel

**Keywords:** HaloTag, Live Imaging, Protein Synthesis

## Abstract

**Background:** HaloTag is a modified bacterial enzyme that binds rapidly and irreversibly to an array of synthetic ligands, including chemical dyes. When expressed in live cells in conjunction with a protein of interest, HaloTag can be used to study protein trafficking, synthesis, and degradation. For instance, sequential HaloTag labeling with spectrally separable dyes can be used to separate preexisting protein pools from proteins newly synthesized following experimental manipulations or the passage of time. Unfortunately, incomplete labeling by the first dye, or labeling by residual, trapped dye pools can confound interpretation.

**Methods**: Labeling specificity of newly synthesized proteins could be improved by blocking residual binding sites. To that end, we synthesized a non-fluorescent, cell permeable blocker (1-chloro-6-(2-propoxyethoxy)hexane; CPXH), essentially the HaloTag ligand backbone without the reactive amine used to attach fluorescent groups.

**Results**: High-content imaging was used to quantify the ability of CPXH to block HaloTag ligand binding in live HEK cells expressing a fusion protein of mTurquoise2 and HaloTag. Full saturation was observed at CPXH concentrations of 5-10 µM at 30 min. No overt effects on cell viability were observed at any concentration or treatment duration. The ability of CPXH to improve the reliability of newly synthesized protein detection was then demonstrated in live cortical neurons expressing the mTurquoise2-HaloTag fusion protein, in both single and dual labeling time lapse experiments. Practically no labeling was observed after blocking HaloTag binding sites with CPXH when protein synthesis was suppressed with cycloheximide, confirming the identification of newly synthesized protein copies as such, while providing estimates of protein synthesis suppression in these experiments.

**Conclusions: **CPXH is a reliable (and inexpensive) non-fluorescent ligand for improving assessment of protein-of-interest metabolism in live cells using HaloTag technology.

## Introduction

The 33 kDa HaloTag protein ^1^ is a modified haloalkane dehalogenase enzyme which bonds covalently to a variety of synthetic ligands (reporters), including chemical dyes. HaloTag dyes do not suffer from some of the drawbacks of traditional fluorescent proteins such as slow tag maturation, tendency to oligomerize, and photoswitching artifacts ^2– 4^. HaloTag fusion proteins can be followed in living cells or *in vivo* for long time durations without label dissociation ^1, 5^. Thus, the HaloTag system represents an attractive method for studying protein localization, dynamics, trafficking, synthesis and degradation. Besides HaloTag, other labeling systems based on similar principles have been developed (e.g. SNAP tag, CLIP tag; ^6– 9^). Compared to SNAP tag, however, the HaloTag system offers superior binding (e.g. 10– 12) and brightness ^13^. Moreover, a wide selection of bright, photostable, cell-permeable HaloTag compatible dyes with rapid labeling kinetics and low nonspecific staining (e.g. 14, 15) is now available.

Both the HaloTag ^1, 4, 16– 20^ and the SNAP tag ^7, 21, 22^ systems have been used with spectrally distinct dyes to visualize newly synthesized proteins, to differentiate between populations of newly formed versus aged proteins, to follow proteins at different subcellular locations and to measure protein half-lifetimes ^23– 31^. In such experiments, however, incomplete labeling – that is, binding sites that remain dye-free, as well as fluorescence from residual unbound ligands – represent significant confounds. Notably, ligands can remain in the cells even after multiple washes (due to slow efflux and reduced active clearance capability, especially in unhealthy cells or at low serum levels (Promega Technical Manual, HaloTag® Technology: Focus on Imaging; see also 32). Moreover, in some cell types, such as cultured neurons, excessive washes can be detrimental ^29, 33^. Saturation of binding sites can be realized by applying fluorescent ligands in large excess (e.g. 34) or Succinimidyl Ester ligands after masking their reactive groups ^32^. This, however, is costly and can introduce other problems, such as the nonspecific labeling mentioned above and even cell toxicity.

Incomplete binding presents a particularly problematic confound when attempting to identify newly synthesized copies of tagged proteins of interest or measure their turnover. Thus for example, 15% unlabeled binding sites, for a protein with a half-life of 5 days, labeled a second time after 24 hours can lead to an erroneous half-life estimate of ~2.5 days, not to mention the misidentification of about half of newly labeled proteins as newly synthesized ones. This confound can be avoided almost entirely by using highly specific, non-fluorescent reagents for blocking residual unbound sites.

Affordable nonfluorescent blockers ^9, 22^ are available for the SNAP tag system. Until most recently, however, there has been a paucity of affordable, nonfluorescent HaloTag compatible blockers. Solutions based on commercially available ligands tend to be costly ^32, 35^ and may not cross the cell membrane efficiently ^36^.

In this study we present an inexpensive, non-fluorescent, cell-permeable HaloTag blocker, 1-chloro-6-(2-propoxyethoxy)hexane, which is well-tolerated both in cell lines and in primary neuronal cultures, and demonstrate its application for following newly synthesized protein using single and dual-color HaloTag labeling.

In the course of this study, four other nonfluorescent compounds were screened as potential HaloTag compatible blockers, of which 7-bromoheptanol was selected as a preferred reagent ^37^. We nevertheless present our findings, in which the characteristics of our alternative blocking reagent and its utility for following protein synthesis in live cells are described, with the hope that it will prove to be useful as well.

All raw images and quantifications are available as *Underlying data* ^38^.

## Results

### A fusion protein for quantifying HaloTag blocking efficacy in living cells

**Figure 1.**
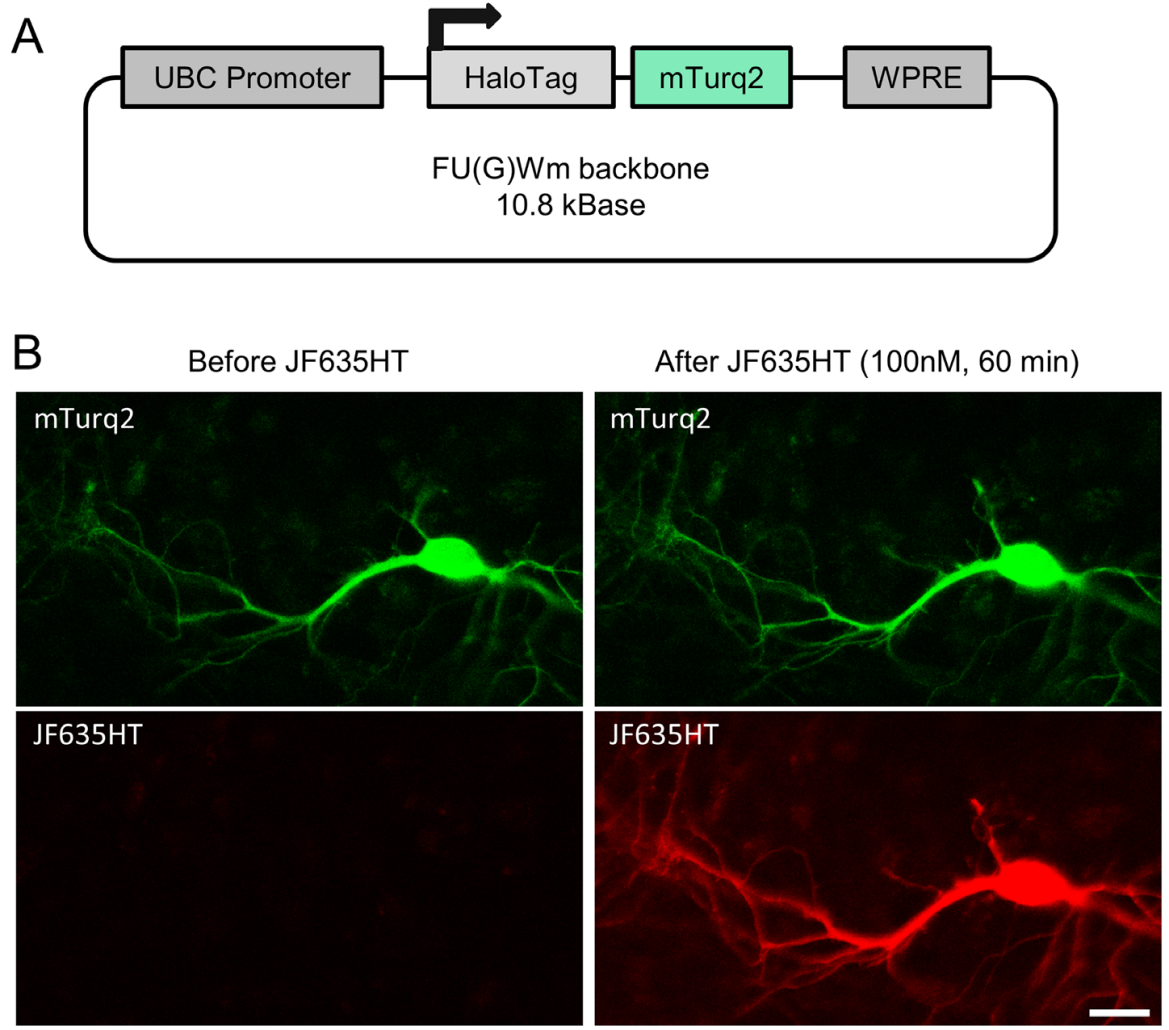
A fusion protein for studying HaloTag labeling and blocker efficacy. ( **A**) A lentivirus-based construct for expressing a HaloTag-mTurquoise2 fusion protein in living cells. UBC = Homo sapiens ubiquitin C; WPRE = woodchuck hepatitis virus posttranscriptional regulatory element. ( **B**) A cortical neuron in primary culture (17 days in culture) expressing the HaloTag-mTurq2 fusion protein, before (left) and after (right) labeling with JF635HT ligand (100nM, 1 hour). Bar, 20µm.

In order to quantify blocking efficacy in living cells, a method was needed to estimate the abundance of HaloTag binding sites (total – free and bound) in individual cells. We reasoned that reporter proteins containing one HaloTag binding site and one copy of a fluorescent protein (FP) would be useful in this regard, as this would allow normalization of HaloTag ligand fluorescence to FP fluorescence and thus calculation of fractional HaloTag labeling values, independent of fusion protein expression levels. To that end, we created a fusion protein of HaloTag and the fluorescent protein mTurquoise2 ^39^ encoded as a single polypeptide (HaloTag-mTurq2; Figure 1A). The DNA coding for the fusion protein was inserted into a third generation lentivirus backbone to allow lentivirus-based expression as well as transfection-based methods (see *Methods*). As shown in Figure 1B, expression of this construct (in rat cortical neurons in culture, in this case) allowed us to simultaneously measure HaloTag ligand binding (Janelia Fluor JF635HT ^15^; a generous gift from Jonathan Grimm and Luke Lavis, Janelia Research Campus) and FP fluorescence in individual cells. As might be expected, no JF635HT binding was observed in neighboring cells devoid of mTurq2 fluorescence, suggesting that HaloTag ligand binding was highly specific. In our hands, neurons expressing HaloTag-mTurq2 could be imaged for many hours (>15) without noticeable toxicity, damage, or negative effects on cell morphology. This fusion construct was thus used in subsequent experiments to quantify the fractional degree of HaloTag ligand binding in live cells.

### Synthesis and efficacy of a non-fluorescent HaloTag blocker

**Figure 2.**
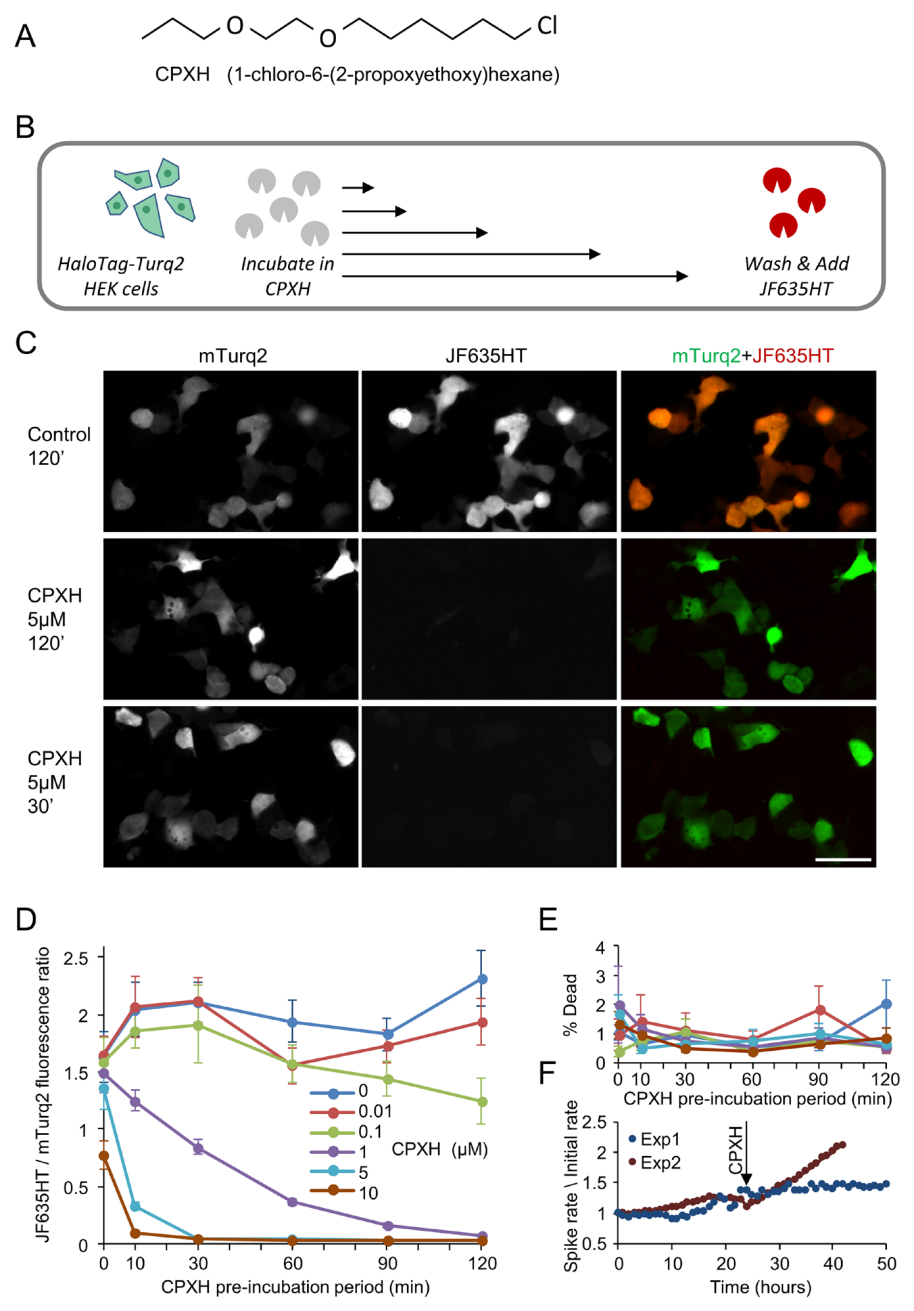
Blocking efficacy of CPXH. ( **A**) Chemical structure of CPXH {1-chloro-6-(2-propoxyethoxy)hexane}, the non-fluorescent, cell-permeable HaloTag blocker tested in this study. ( **B**) Testing CPXH blocking efficacy. HaloTag-mTurq2 was expressed in HEK293 cells growing in 96 well plates. The cells were treated with 0.01, 0.1 ,1 ,5, or 10 µM CPXH, or carrier solution (0.1% DMSO in cell culture media), for 10, 30, 60, 90, or 120 minutes (all combinations of concentration and duration). The cells were washed with cell culture media and labeled with JF635HT (100nM for 30 minutes). The cells were then washed with HBSS and imaged. Imaging was carried out on an automated high-content imaging system, providing reads from >10,000 cells per concentration and time point per experiment (2 separate experiments). Note that cells at t=0 were treated with CPXH and washed immediately, and were thus exposed to the blocker for anywhere between a few seconds and a minute. ( **C**) Representative images of JF635HT labeling in mTurq2-positive HEK293 cells. Cells were exposed to CPXH (or carrier solution) at indicated concentrations and durations. Bar, 50µm. ( **D**) JF635HT / mTurq2 (background-corrected) fluorescence ratios at the CPXH concentrations and incubation times tested. Only fields of view with at least 90 mTurq2 positive cells were included (8 to 16 fields of view per condition, two separate experiments). Systematic increases in blocking efficacy were observed with increases in CPXH concentrations and pre-incubation durations. Slight increases in JF635HT / mTurq2 ratios were also observed following pre-incubation with carrier solution or low CPXH concentrations, which might relate to the presence of DMSO in the carrier solution. ( **E**) Live/Dead assays (using Propidium Iodide / NucBlue Live, HEK293 cells, and high-content imaging as in **C**, **D**) point to negligible cell death at all tested CPXH concentrations and treatment durations with no dependence on concentration or duration. ( **F**) CPXH (10 µM) did not suppress spontaneous network activity (a sensitive measure of neuronal viability) in networks of rat cortical neurons growing on multielectrode arrays. CPXH was added after 24 hours of baseline recordings, and activity was followed for additional 18 or 47 hours (two experiments). Spike rates (action potentials recorded from all electrodes per minute; one-hour averages) were normalized to spike rates during the first hour (1,546 and 7,663 spikes/min, respectively). Error bars ( **D, E**) indicate SEM of fields of view.

Most HaloTag ligands are based on a chlorinated alkyl chain with a reactive group for attaching fluorescent probes on the opposite side. Here, we synthesized the same ligand without the reactive group, reasoning that its binding properties would be similar but its ability to move across cell membranes improved, and its non-specific reactivity reduced, by the removal of the terminal polar amine group. The resulting molecule {(1-chloro-6-(2-propoxyethoxy)hexane; CID 63684368)} referred to here as CPXH is shown in Figure 2A.

To test the efficacy of CPXH as a non-fluorescent HaloTag blocker, and characterize its useful concentrations and blocking kinetics, we expressed HaloTag-mTurq2 in HEK293 cells, and treated the cells with 0.01, 0.1, 1, 5, or 10 µM of CPXH, or carrier solution, for 10, 30, 60, 90, or 120 minutes (all combinations of treatment durations and concentrations). The cells were then washed and labeled with JF635HT at a final concentration of 100 nM for 30 minutes ( Figure 2B). Imaging was carried out on an automated high-content imaging system, providing reads from >10,000 cells per concentration and time point per experiment (two separate experiments). Background corrected JF635HT/mTurq2 fluorescence ratios were then calculated for all concentrations and durations. As shown in Figure 2C, D, 30-minute exposures to 5 or 10 µM CPXH completely prevented labeling with JF635HT, suggesting saturation of practically all HaloTag binding sites. Nearly full saturation was also attained following exposure to 1 µM CPXH for 120 min.

To examine the potential cytotoxicity of CPXH, HEK293 cells were treated with CPXH at a concentration of 0.01, 0.1, 1, 5, or 10 µM or carrier solution, for 10, 30, 60, 90, or 120 minutes and cell viability was tested using a Live/Dead assay (see *Methods*) and high-content screening microscopy. Cell death was found to be negligible at all concentrations and treatment durations tested (0.89±0.44%, average ± standard deviation; maximum ~2% cell death) with no dependence whatsoever on CPXH concentration or exposure duration ( Figure 2E). Furthermore, in networks of rat cortical neurons grown on multielectrode arrays ^40^, levels of spontaneous network activity - a sensitive measure of neuronal viability - did not decline following chronic exposure to 10 µM CPXH for 18 and 47 hours ( Figure 2F; two experiments). These findings suggest that CPXH is a non-toxic, efficient blocker of HaloTag binding sites.

### Using HaloTag and CPXH to follow newly synthesized proteins

**Figure 3.**
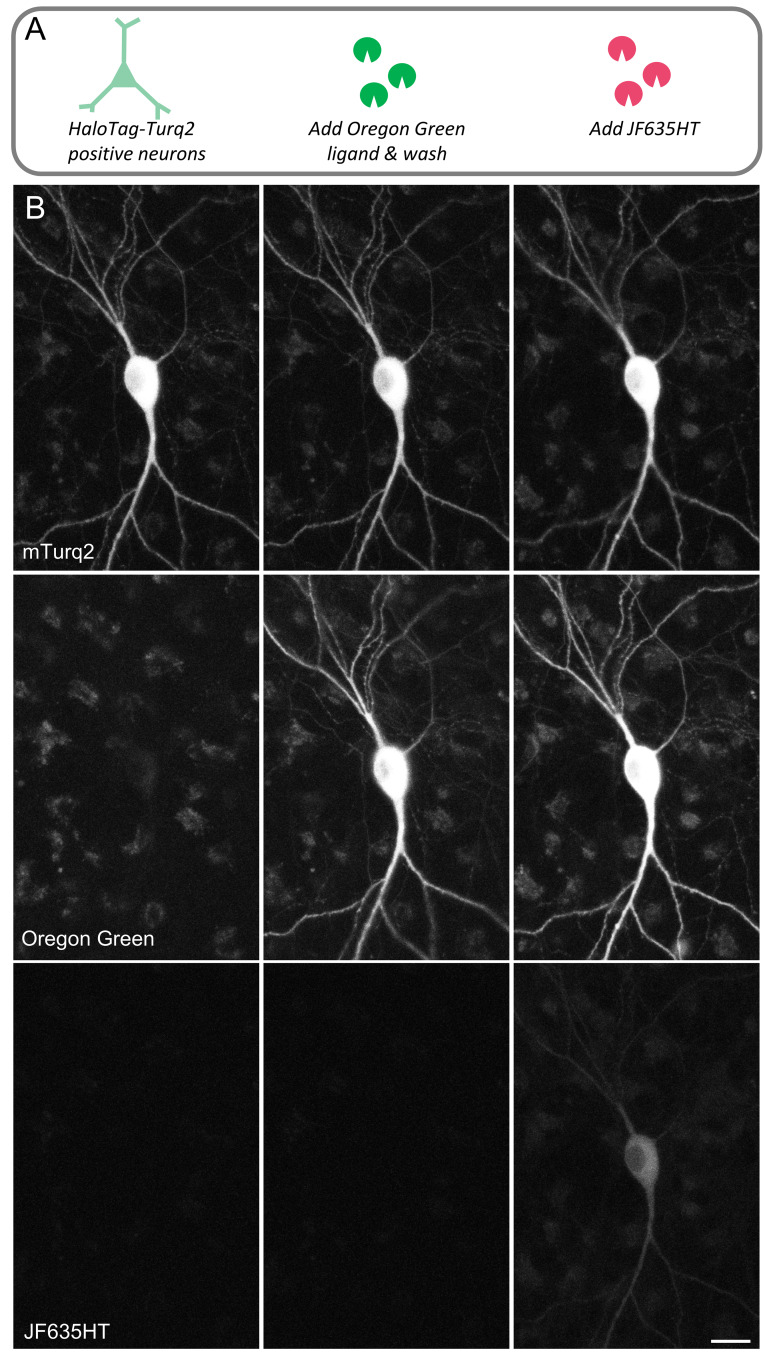
Significant residual free binding sites after conventional labeling with fluorescent HaloTag ligand. ( **A**) mTurq2-positive neurons in cell culture were labeled with Oregon Green HaloTag ligand (Promega 100nM, 1 hour), washed, and labeled immediately with JF635HT ligand. ( **B**) Applying JF635HT resulted in significant labeling, indicating incomplete saturation of HaloTag binding sites. JF635HT labeling occurred also when using a 10-fold higher concentration of Oregon Green (Promega 1 µM, 1hour; not shown). Bar, 20µm.

An exciting application of HaloTag technology concerns the labeling of newly synthesized copies of proteins of interest and following their fates thereafter. As explained in the Introduction, correct interpretation of such experiments necessitates the complete labeling of all HaloTag binding sites in preexisting protein copies. In preliminary experiments, and in agreement with prior studies ^32, 37^ we noted that applying a second fluorescent HaloTag ligand (Oregon Green, Promega, or JF635HT) immediately following apparently saturating labeling with a first ligand (JF635HT or Oregon Green, Promega) still resulted in significant labeling with the second ligand, indicating incomplete saturation of HaloTag binding sites, as exemplified in Figure 3.

**Figure 4.**
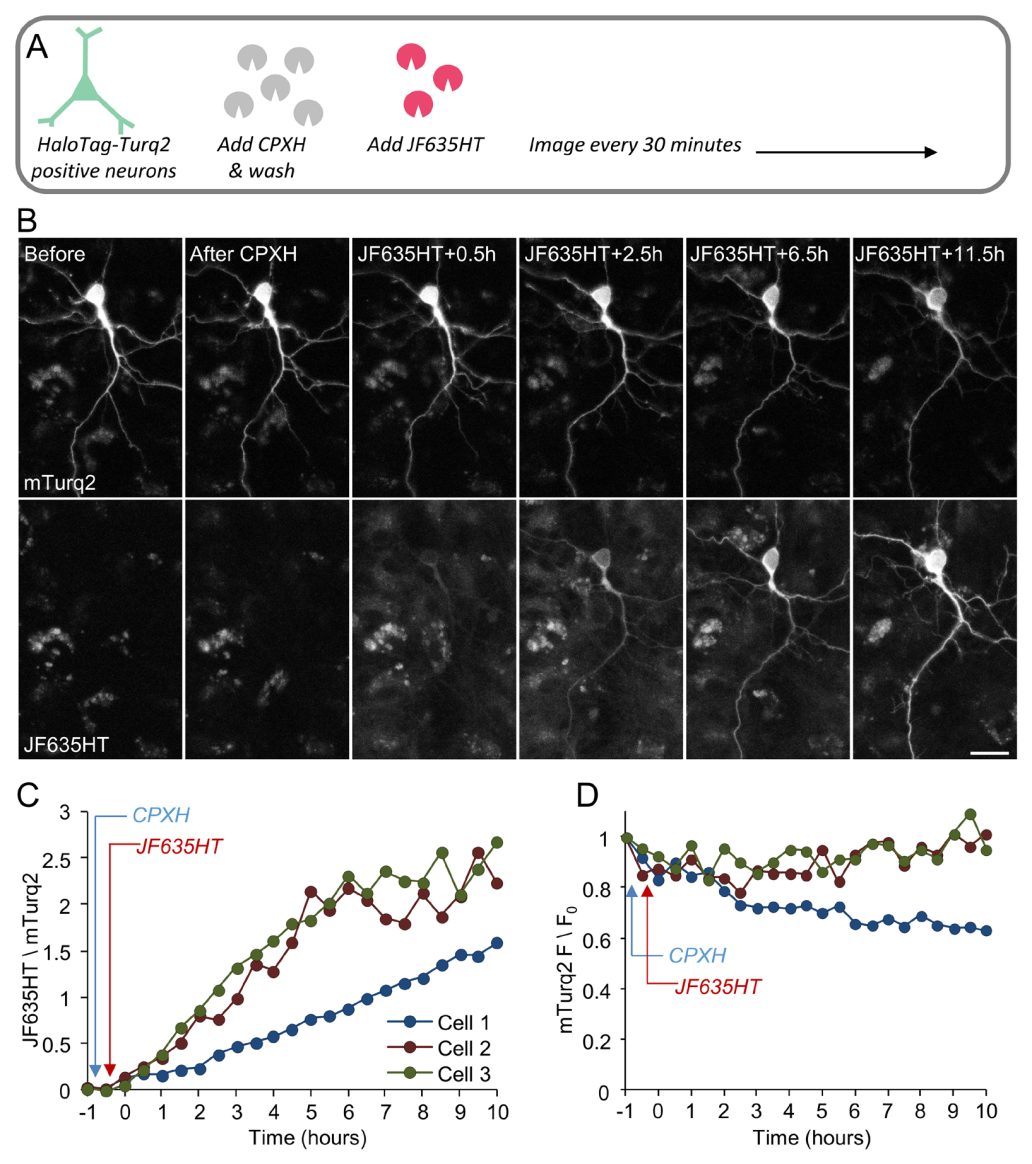
CPXH improves the reliability of newly synthesized protein detection in living cells. ( **A**) Neurons expressing HaloTag-mTurq2 were first treated with CPXH (10 µM) for 30 minutes. The cells were then washed, followed by addition of JF635HT to the cell culture media (100nM, without washing it out), and the cells were followed by time-lapse imaging. ( **B**) Example of mTurq2-positive cortical neuron (17 days in culture) treated as described in A and followed by time lapse imaging. Bar, 20µm. ( **C**) Changes in JF635HT labeling over time were quantified by measuring, on a cell-by-cell basis, the ratio of JF635HT to mTurq2 fluorescence in mTurq2-positive cells. Background corrected JF635HT/mTurq2 fluorescence ratios for three different neurons are shown. Images in B belong to Cell 1. Note the negligible labeling immediately after exposure to JF635HT (compare with Figure 3B). ( **D**) mTurq2 fluorescence in the same cells, expressed as a fraction of each cells initial fluorescence.

**Figure 5.**
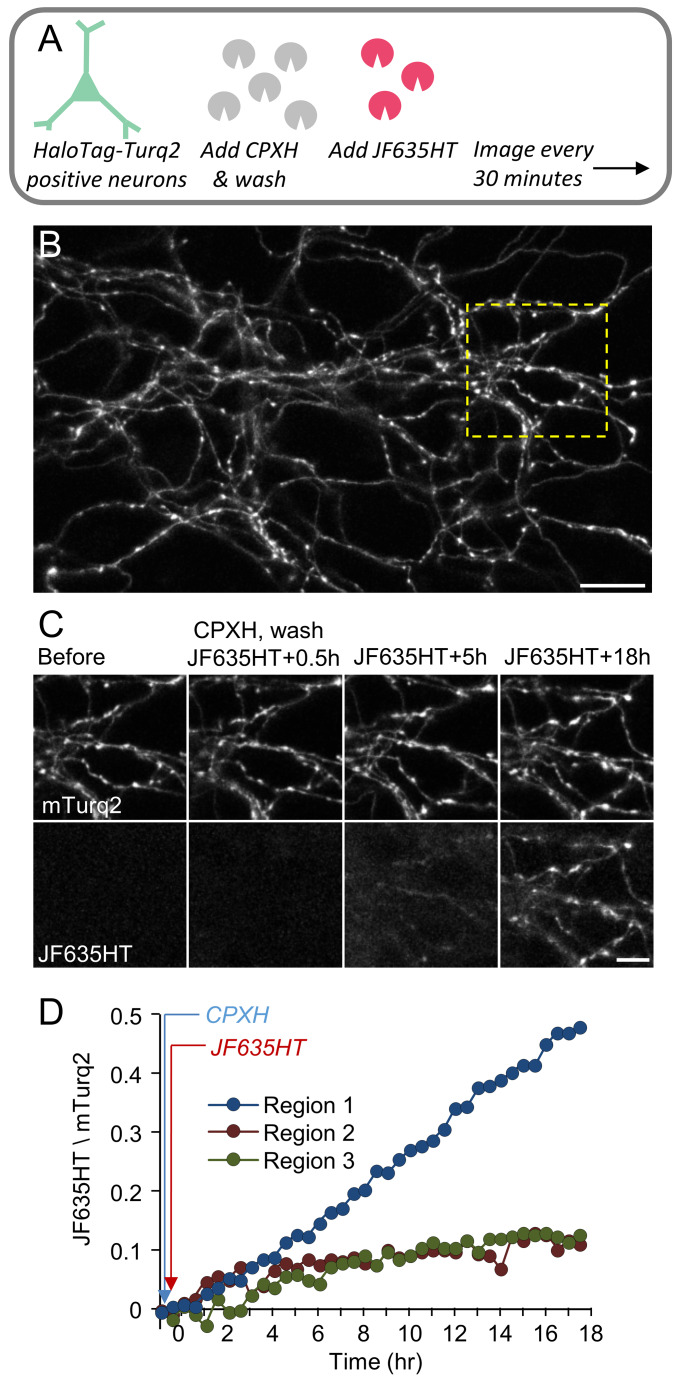
CPXH improves the reliability of newly synthesized protein detection in axons of living neurons. ( **A**) Imaging of axon ‘beds’ belonging to neurons expressing HaloTag-mTurq2. Neurons were treated as in Figure 4. ( **B**) Example of axon bed belonging to mTurq2-positive cortical neuron(s). Bar, 20 µm. ( **C**) Gradual changes in JF635HT labeling over time in axons enclosed in the yellow rectangle in **B**. Note the negligible labeling immediately after exposure to JF635HT. Bar, 10 µm. ( **D**) ratio of JF635HT to mTurq2 fluorescence in mTurq2-positive axons (average of 10 regions per field of view). Background corrected JF635HT/mTurq2 fluorescence ratios for three different axon beds are shown. Images in ( **B**) and ( **C**) belong to Region 1. Note the slow increase in JF635HT fluorescence, as might be expected in cellular regions at significant distance from the neurons’ major sites of protein synthesis (i.e., the cell bodies).

**Figure 6.**
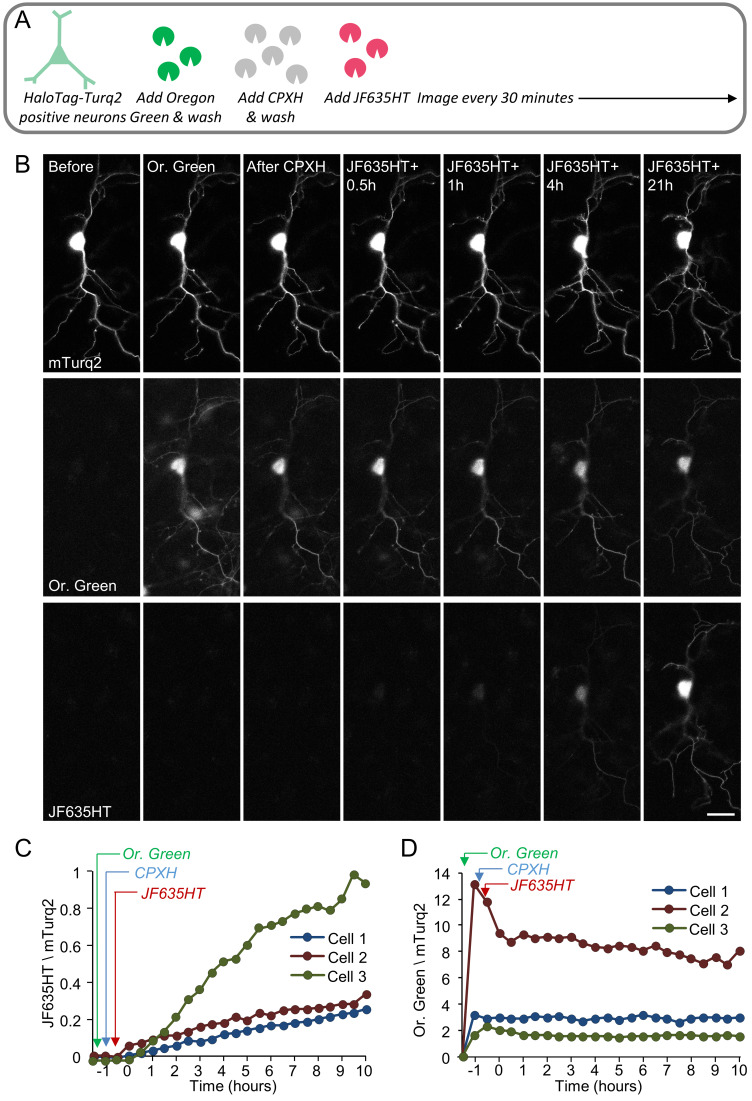
Dual-color labeling time-lapse of neurons expressing HaloTag-mTurq2. ( **A**) Neurons expressing HaloTag-mTurq2 were labeled with Oregon Green ligand (Promega 1 µM, 30 min), washed, and blocked with CPXH (10 µM) for 30 minutes. The cells were then washed and labeled with JF635HT and followed by time-lapse imaging. ( **B**) Example of mTurq2-positive cortical neuron (14 days in culture) treated as described in A and followed by time lapse imaging. Bar, 20µm. ( **C**) Changes in JF635HT, and ( **D**) Oregon Green labeling over time were quantified by measuring, on a cell-by-cell basis, the ratio of JF635HT (or Oregon Green) to mTurq2 fluorescence in mTurq2-positive cells. Background corrected fluorescence ratios for three neurons are shown. Images in B belong to Cell 1.

As a proof of principle, we examined whether unambiguous identification of newly synthesized proteins would be facilitated by using CPXH to block residual binding sites. To that end, we performed single and dual-labeling time-lapse experiments of neurons expressing HaloTag-mTurq2, using CPXH at concentrations derived from the experiments in HEK293 cells ( Figure 2) to block residual binding sites before applying the (second) label. In the first set of experiments ( Figure 4 and Figure 5), neurons expressing HaloTag-mTurq2 were first treated with CPXH (10 µM) for 30 minutes. The cells were then washed, JF635HT was added to the cell culture media (without washing it out) and the cells were followed by time-lapse imaging for >12 hours. In a second set ( Figure 6), blocking with CPXH was preceded by labeling with Oregon Green ligand (Promega). Changes in JF635HT (and Oregon Green) labeling over time were quantified by measuring, on a cell-by-cell basis, the ratio of JF635HT (or Oregon Green) to mTurq2 fluorescence in mTurq2-positive cells. To correct for nonspecific label accumulation, fluorescence values in neighboring mTurq2-negative areas were obtained at each time point and subtracted from JF635HT / Oregon Green fluorescence in mTurq2-positive cells. As shown in Figure 4C, Figure 5C and Figure 6C, initial JF635HT labeling was negligible; with time, however, JF635HT fluorescence gradually increased, probably reflecting the labeling of newly synthesized HaloTag-mTurq2 copies. This was observed for cell bodies and dendrites ( Figure 4 and Figure 6) as well as distal axon ‘beds’ ( Figure 5), albeit at slower rates, as might be expected for newly synthesized protein copies delivered from remote somatic protein synthesis facilities. Interestingly, we noted that labeling with Oregon Green ligand was associated with strong, non-specific labeling in all cells (mTurq2-positive and negative alike) that tended to wash out relatively slowly (over a few hours), in particular from cell bodies and thick neuronal processes. The slow efflux of this ligand (probably due to HaloTag Oregon Green ligand deacetylation; Technical Manual, HaloTag Technology) almost certainly underlies the initial decay of Oregon Green fluorescence ( Figure 6D). In contrast, very low levels of non-specific JF635HT labeling were observed even after prolonged periods in the presence of this ligand. A second potential confound concerns the constancy of mTurq2 fluorescence (used here to normalize HaloTag ligand fluorescence). As shown in Figure 4D, a small decline was observed in some, but not all neurons, probably due to photobleaching (~13±16%, average ± standard deviation, 10 hours for all cells shown in Figure 4– Figure 6) indicating that absolute JF635HT/mTurq2 increase rates might have been slightly overestimated.

These experiments thus suggest that CPXH can greatly facilitate unambiguous identification of newly synthesized proteins in live cells and through continuous HaloTag labeling.

### Labeling following CPXH blocking when protein synthesis is suppressed

**Figure 7.**
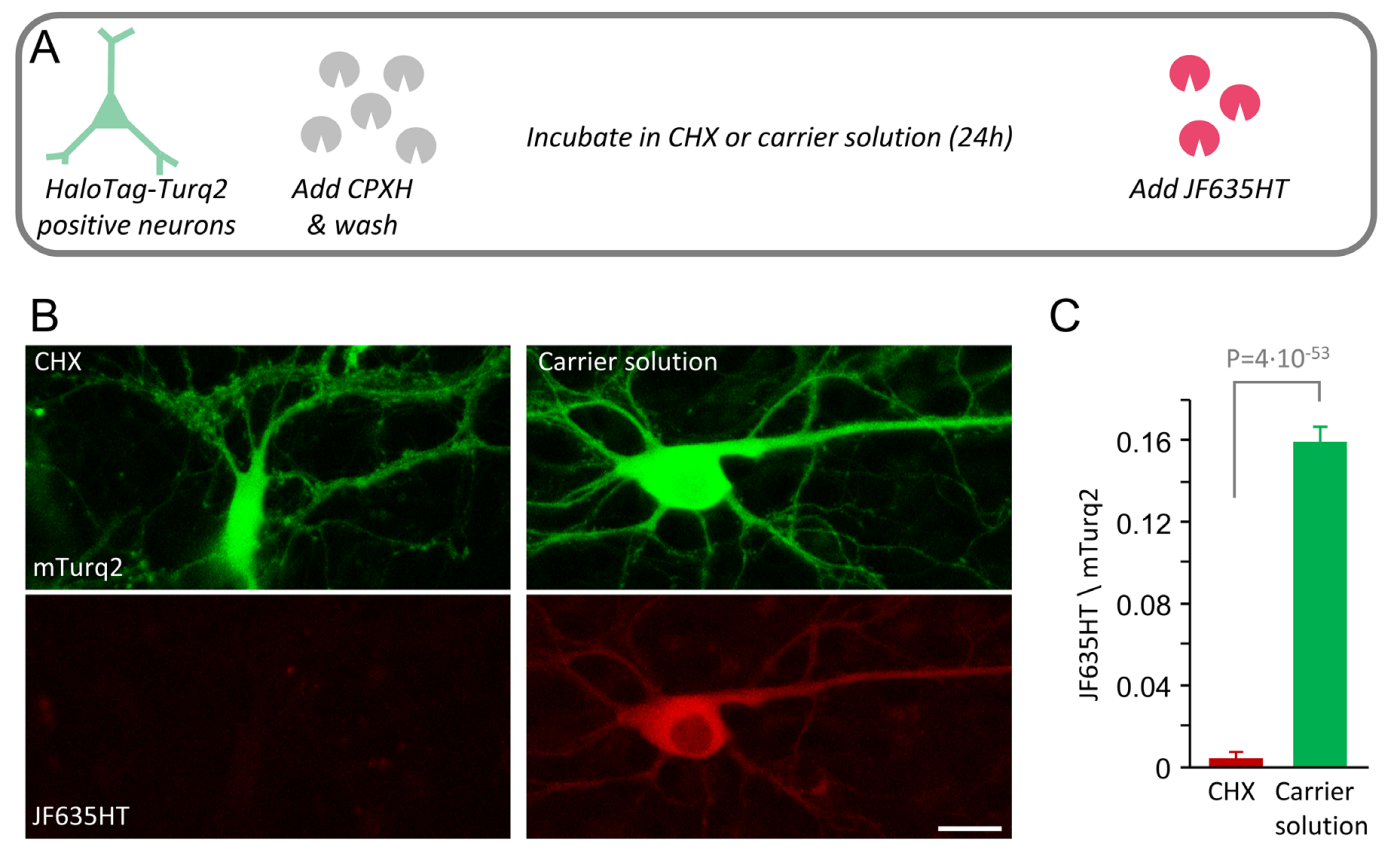
Proteins labeled with fluorescent HaloTag ligands following CPXH blocking reflect newly synthesized protein copies. ( **A**) Cortical neurons expressing HaloTag-mTurq2 were treated with CPXH. The cells were washed and thereafter exposed to cycloheximide (CHX) or carrier solution (0.1% DMSO in cell culture media) for 24 hours, followed by JF635HT labeling. ( **B**) JF635HT labeling in two such neurons (left: 24-hour CHX; right: 24-hour carrier solution). Scale bar 20µm. ( **C**) 24-hour protein synthesis suppression resulted in a nearly 40-fold (39.2) reduction in JF635HT labeling intensity. There were 81 and 178 neurons, CHX and carrier solutions, respectively, 4 replicates per condition from 2 separate experiments. Average + SEM, t-test assuming unequal variances.

The accumulation of JF635HT in the experiments of Figure 4– Figure 6 presumably reflected the accumulation of newly synthesized HaloTag-mTurq2. To validate this assumption, we examined JF635HT labeling after cells were blocked with CPXH and then exposed to the potent protein synthesis inhibitor cycloheximide (CHX; 100 µg/ml). Specifically, cortical neurons expressing HaloTag-mTurq2 were treated with CPXH as described above. The cells were then washed and exposed to CHX or carrier solution for 24 hours, followed by JF635HT labeling. As shown in Figure 7, practically no labeling was observed following the 24-hour exposure to CHX; In fact, quantification revealed a nearly 40-fold reduction in labeling intensity ( Figure 7C). These findings support the aforementioned presumption, and, at the same time, provide a quantitative assessment of protein synthesis suppression by CHX in these preparations.

## Discussion and conclusions

The findings described above suggest that CPXH is a potent non-fluorescent, cell-permeable, and non-toxic HaloTag ligand. We first characterized its blocking kinetics and their dependence on CPXH concentration. We then demonstrated that CPXH can improve the fidelity of experiments aimed at following newly synthesized proteins in living cells. Finally, we show how this blocker might be useful for quantifying protein synthesis inhibitor efficacy in living cells.

A recent study ^37^ identified 7-bromoheptanol as an alternative low-toxicity HaloTag-blocking agent, and demonstrated its usefulness for measuring protein turnover at the population and single cell level. The authors suggested that this agent could be potentially used for estimating the synthesis rate of proteins of interest within individual cells. The experiments shown here ( Figure 4– Figure 6) confirm this suggestion. Moreover, the use of the HaloTag-mTurq2 fusion protein in our experiments provided confidence that the gradual labeling observed over time in mTurq2-positive cells reflected *bona-fide* HaloTag labeling, and not, e.g., non-specific accumulation of labels in cells, while providing means for normalizing HaloTag labeling to total numbers of HaloTag binding sites. We note that normalization to mTurq2 levels in such experiments might be somewhat imperfect, as new mTurq2 is synthesized alongside new HaloTag binding sites. Yet at least at initial time points, the contribution of newly synthesized mTurq2 to total mTurq2 fluorescence is probably insignificant, even less so if total HaloTag-mTurq2 levels remain more or less constant. At later time points, however, the latter assumption was not always valid, as mTurq2 fluorescence declined slightly in some neurons, possibly due to mTurq2 photobleaching. Thus, quantitative assessments of protein synthesis rates based on this approach will require corrections for these potential confounds, as well as others, such as ligand photobleaching, efflux and unbinding, as well as HaloTag and FP maturation kinetics.

The affinity, cell entry, binding or washout kinetics of CPXH were not measured here or compared with those of 7-bromoheptanol, and thus their advantages and disadvantages with respect to each other remain unknown. Given that the structure of CPXH is essentially identical to the backbone of most HaloTag ligands, it might be expected to be as affine and effective as the commonly used fluorescent ligands themselves. Hopefully, future studies will provide further information on these and other agents that optimize the utility of HaloTags and their like.

## Methods

### Nonfluorescent HaloTag blocker CPXH

HaloTag blocker 1-chloro-6-(2-propoxyethoxy)hexane (CID 63684368) was synthesized at our request by AKos Consulting & Solutions, GmbH. The dry material was dissolved in DMSO to prepare a 10 mM stock solution, which was stored in small aliquots at -20°C until used.

### HaloTag-mTurquoise2 construct

FU-HaloTag-mTurq2-Wm was generated based on FU-PSD95-mTurq2-Wm ^41^. Briefly, AgeI-HaloTag-XhoI (911 bp; sequence below) was synthesized *de novo* based on the Halotag sequence from Promega pFN23A HaloTag® CMVd2 Flexi® Vector, 9PIG286 ( https://worldwide.promega.com/-/media/files/vector-sequences/flexi/pfn23a.txt). FU-PSD95-mTurq2-Wm was cut with AgeI and XhoI and the PSD-95 segment was removed, replaced by AgeI-HaloTag-XhoI and ligated to obtain FU-HaloTag-Turq2-Wm. Cloning and custom gene synthesis was done by Genscript (Piscataway NJ, US). Positioning the HaloTag at the N-terminus was based on the literature (e.g. 42) and on the structure of the protein of interest.

AgeI-HaloTag-XhoI:

ACCGGTCGCCACCATGGCAGAAATCGGTACTGGCTTTCCATTCGACCCCCATTATGTGGAAGTCCTGGGCGAGCGCATGCACTACGTCGATGTTGGTCCGCGCGATGGCACCCCTGTGCTGTTCCTGCACGGTAACCCGACCTCCTCCTACGTGTGGCGCAACATCATCCCGCATGTTGCACCGACCCATCGCTGCATTGCTCCAGACCTGATCGGTATGGGCAAATCCGACAAACCAGACCTGGGTTATTTCTTCGACGACCACGTCCGCTTCATGGATGCCTTCATCGAAGCCCTGGGTCTGGAAGAGGTCGTCCTGGTCATTCACGACTGGGGCTCCGCTCTGGGTTTCCACTGGGCCAAGCGCAATCCAGAGCGCGTCAAAGGTATTGCATTTATGGAGTTCATCCGCCCTATCCCGACCTGGGACGAATGGCCAGAATTTGCCCGCGAGACCTTCCAGGCCTTCCGCACCACCGACGTCGGCCGCAAGCTGATCATCGATCAGAACGTTTTTATCGAGGGTACGCTGCCGATGGGTGTCGTCCGCCCGCTGACTGAAGTCGAGATGGACCATTACCGCGAGCCGTTCCTGAATCCTGTTGACCGCGAGCCACTGTGGCGCTTCCCAAACGAGCTGCCAATCGCCGGTGAGCCAGCGAACATCGTCGCGCTGGTCGAAGAATACATGGACTGGCTGCACCAGTCCCCTGTCCCGAAGCTGCTGTTCTGGGGCACCCCAGGCGTTCTGATCCCACCGGCCGAAGCCGCTCGCCTGGCCAAAAGCCTGCCTAACTGCAAGGCTGTGGACATCGGCCCGGGTCTGAATCTGCTGCAAGAAGACAACCCGGACCTGATCGGCAGCGAGATCGCGCGCTGGCTGTCGACGCTGGAGATTTCCGGCGCTCGAG

### HaloTag ligands

Janelia Fluor 635 HaloTag ligand (JF635HT ^15^; 100 nM) was incubated for 30 minutes (or 1 hour; experiment of Figure 3) at 37°C, 5% CO _2_. Oregon Green cell permeable ligand (100nM for 1 hour / 1 µM for 30 minutes; experiments of Figure 3 and Figure 6, respectively; Promega) was applied at 37°C, 5% CO _2_, then repeatedly washed to remove unbound ligand.

### Cell lines

HEK293 cells were grown in DMEM supplemented with 10% FBS at 37°C, 5% CO _2_. The cells were transfected with HaloTag-mTurq2 using Calfectin (Signagen) transfection according to the manufacturer’s protocol. In brief, one hour before transfection the media was replaced with complete medium with serum and antibiotics. HaloTag-mTurq2 DNA was diluted in serum-free, high glucose DMEM. Calfectin reagent was added to the tube, and pipetted 3–4 times to mix. The mixture was incubated for 10–15 minutes, and applied dropwise to the cells. The plate was swirled gently to mix and returned to the incubator. At 12–18 hours following transfection the media was replaced with fresh culture media. HEK293 cells were reseeded into 96-well glass-bottom microplates (µClear, Black; Greiner) 24 hours after transfection (20k cells per well). At 24 hours after reseeding, the cells were washed twice in Hanks Balanced Salt Solution (HBSS).

### Primary cultures of rat cortical neurons

Primary cultures of rat cortical neurons were prepared as described previously ^43^ using a protocol approved by the Technion committee for the supervision of animal experiments (IL-116-08-71). Briefly, cortices of two 1-2-day-old Wistar rats of either sex (Charles River, UK) were dissected following rapid decapitation, dissociated by trypsin treatment followed by trituration using a siliconized Pasteur pipette, and plated onto 22×22 mm coverslips coated with polyethylenimine (Sigma) inside 8-mm-diameter glass cylinder microwells (Bellco Glass). Cells were initially grown in medium containing Minimum Essential Medium (MEM; Sigma), 25 mg/l insulin (Sigma), 20 mM glucose (Sigma), 2 mM L-glutamine (Sigma), 11 mg/l gentamycin sulfate (Sigma), and 10% NuSerum (Becton Dickinson Labware). The preparation was then transferred to a humidified tissue culture incubator and maintained at 37°C in a 95% air and 5% CO _2_ mixture. Half the volume of the culture medium was replaced every 7 days with cell culture medium similar to the medium described above but devoid of NuSerum, containing a lower concentration of L-glutamine (Sigma, 0.5 mM), and 2% B-27 supplement (Gibco). Neurons used in these experiments came from approximately 20 separate preparations. HaloTag-mTurq2 DNA was expressed by calcium phosphate transfection, or by addition of HaloTag-mTurq2 lentiviral particles to the neuronal cultures (as detailed below).

### Calcium phosphate transfection

Cortical neurons were transfected with HaloTag-mTurq2 by calcium phosphate transfection 9–11 days after plating, as described previously ^44^. Neurons were imaged within the cloning cylinders 14–21 days after plating, in the cell culture media.

### Lentivirus production and lentiviral transduction

HaloTag–mTurq2 lentiviral particles were produced in house using a commercially available kit (ViraPower™, ThermoFisher Scientific). Briefly, 80%-confluent HEK293T cells were transfected using Lipofectamine 2000 (Invitrogen) with a mixture of three Virapower kit packaging plasmids (pLP1, pLP2, pLP/VSVG), and the expression vector. Lentiviral stocks were prepared by collecting the supernatant 48 hours after transfection, filtering by a 0.45-μm filter, and storing the liquid in small aliquots at -80°C. To express the DNA in cultures of neuronal cells, 4 days after plating the neurons, 1.5 μl of HaloTag-mTurq2 virus were added to each of the cortical neuronal cultures. Neurons were imaged within the cloning cylinders 14–21 days after plating, in the cell culture media.

### Protein synthesis inhibition experiments

Experiments were carried out on neurons grown in culture for 2.5 to 3 weeks. The original cell culture media was set aside and CPXH was applied for 30 minutes (10 µM in cell culture media). The cells were then washed three times in cell culture media and the original media was returned. Cycloheximide (100 µg/ml, Sigma) or carrier solution was then applied and the cells were returned to a 37°C, 5% CO _2_ incubator. 24 hours later, JF635HT was added to the cells for 30 minutes, and the cells were imaged immediately.

### Cell viability testing

Propidium Iodide and NucBlue Live reagent (Hoechst 33342) staining (ReadyProbes Cell Viability Imaging Kit Blue/Red; Thermo) were applied according to the manufacturer’s instructions to assess cell viability in HEK293 cells. The cells were treated with different blocker concentrations (0.01, 0.1, 1, 5, 10 µM, or carrier solution) for a range of time durations (0, 10, 30, 60, 90, or 120 minutes). After treatment with the blocker, the cells were washed twice in HBSS. The two reagents were applied (two drops per ml of each reagent); NucBlue was applied for 15 minutes, and Propidium Iodide was applied just before imaging (for less than 10 minutes). The cells were washed with HBSS, and imaged. Nine sites (3×3 grid) per well were automatically selected at fixed positions relative to the microplate. Total cell count per field of view (NucBlue positive cells) and dead cell count per field of view (cells stained positive for both NucBlue and Propidium Iodide) were calculated by the imaging system’s integrated software. Cell viability in each well was assayed by calculating the average ratio (from nine fields of view) of dead cell count to total cell count for each concentration and duration. Only fields of view with at least 50 NucBlue-positive cells were included.

### Microscopy and image analysis

Neuronal cultures were imaged on a custom-designed confocal laser scanning microscope ^43^ using a 40X, 1.3 NA Fluar objective. Images were collected by averaging six frames at three focal planes spaced 0.8 µm apart. All data were collected at a resolution of 640×480 pixels, at 12 bits per pixel. Excitation of mTurq2 was performed at 457 nm. Emission was read using either 467-493 nm or 465-485 band pass filters. Excitation of JF635HT was performed at 633 nm. Emission was read using a 635 nm long-pass filter (Semrock). Excitation of Oregon Green was performed at 488nm. Emission was read using a 500–550 nm band pass filter (Chroma).

Image analysis was performed using custom-written software (“OpenView”; available on request; analyses could also be performed using other software packages, such as ImageJ) by placing rectangular regions of interest on cell bodies and dendrites (4 to 7 regions per cell, Figure 4 and Figure 6), axons (10 regions, Figure 5), or cell bodies ( Figure 7) of mTurq2-positive neurons using the mTurq2 channel. Fluorescence values of all channels were then collected from these regions and average values were calculated for each neuron or axonal bed. To correct for background fluorescence and non-specific labeling, regions of interest were placed on mTurq2-negative areas ( Figure 4– Figure 6) or mTurq2-negative somata ( Figure 7) and these values were subtracted from all fluorescence readings for all channels and all time points.

Imaging of HEK293 cells was done in a 5% CO _2_, temperature-controlled 37°C microscope chamber of a high-content imaging system (ImageXpress, Molecular Devices). The cells were washed with HBSS before imaging. A single Z-section (2048×2048 pixels, 16 bits per pixel) was obtained automatically at each site using a 20x objective and the system’s sCMOS camera. For non-fluorescent blocker efficacy measurements, 25 sites (a 5×5 grid, automatically selected) were imaged in each well. mTurq2 images were acquired using the following filter set: 438/29nm (ex.), 458nm (dichroic), 483/32nm (em.); JF635HT images were acquired using the following filter set: 562/40nm (ex.), 593nm (dichroic), 641nmLP (em.). For the live-dead assay, nine sites (3×3 grid) per well were automatically selected at fixed positions relative to the microplate using the following filters sets for NucBlue (377/50 nm, 409 nm, 447/60 nm,) and propidium iodide (562/40 nm, 593 nm, 624/40 nm; ex., dichroic, em., respectively). Data analysis was performed automatically using the imaging system’s integrated software, and data were thereafter exported to Microsoft Excel for further analysis.

Images for figures were processed by uniform contrast enhancement and low-pass filtering using Adobe Photoshop and prepared for presentation using Microsoft PowerPoint.

## Data availability

### Underlying data

Figshare: A non-fluorescent HaloTag blocker for improved measurement and visualization of protein synthesis in living cells. https://doi.org/10.6084/m9.figshare.12115509.v1 ^38^.

This project contains the following underlying data:

- Cohen Boulos Ziv Data Fig 1 (ZIP). (Image data underlying Figure 1.)
- Cohen Boulos Ziv Data Fig 2 (ZIP). (Image data and quantification of blocking efficacy underlying Figure 2.)
- Cohen Boulos Ziv Data Fig 3 (ZIP). (Image data underlying Figure 3.)
- Cohen Boulos Ziv Data Fig 4 (ZIP). (Image data underlying Figure 4.)
- Cohen Boulos Ziv Data Fig 5 (ZIP). (Image data and fluorescence quantification underlying Figure 5).
- Cohen Boulos Ziv Data Fig 6 (ZIP). (Image data and fluorescence quantification underlying Figure 6.)
- Cohen Boulos Ziv Data Fig 7 (ZIP). (Image data and fluorescence quantification underlying Figure 7.)

Data are available under the terms of the Creative Commons Attribution 4.0 International license (CC-BY 4.0).
