# Supplementary material for: A non-fluorescent HaloTag blocker for improved measurement and visualization of protein synthesis in living cells
Source: F1000Res. 2020 Jun 8;9:ISF-302. Originally published 2020 Apr 28. [Version 2] doi: 10.12688/f1000research.23289.2 (PMC7255903; doi:10.12688/f1000research.23289.2)
Supplement: Peer review history

# Peer review: Reviewer response for version 1

**DOI:** 10.5256/f1000research.25710.r62881

**Published:** 2020-05-26

**Authors:** Susanne tom Dieck

**Affiliations:** 1 Synaptic Plasticity Department, Max Planck Institute for Brain Research, Frankfurt am Main, Germany

In this manuscript Cohen *et al.* describe a valuable addition to the HaloTag toolbox. They developed and characterized an efficient and inexpensive non-fluorescent blocker (ligand) of HaloTag sites. They make use of a fluorescent protein/HaloTag fusion-based experimental design to evaluate relative labeling of HaloTag sites with fluorescent ligands offering the advantage to normalize and also quantify background labeling. As a result they present a strategy to robustly identify and label newly synthesized HaloTag proteins without several current pitfalls.

 The need of such a low-cost, efficient blocker is underlined by the simultaneous development of a similar compound in a different lab (as properly cited). 

 The manuscript without doubt describes a very useful compound and method in sufficient detail to be used by others as it is. As an improvement, a couple of small points could be discussed in more detail to guide the reader through potential further uses, pitfalls or limitations or could be presented with more of the available information embedded into the figures:

 Related to Figure 2:

- As important for the method in the text regarding the quantification in D the points of ‘complete’ blocking are discussed. It nonetheless would be helpful to also have a sentence on the data without and with low concentration of CPXH (e.g. high variability between time points in the 0µM).
- Add a line how similar this is for neurons (was the concentration used in the following experiments based on the HEK data or determined independently).
- Toxicity of new compounds and also influence on physiology is an important issue (especially in neurons). The authors have addressed this by not only live/dead assays in HEK cells but also generated data by long-term CPXH incubation on neuronal network activity. This important dataset should be present in a main figure.

Related to Figure 4-6/discussion:

- The use of a fluorescent protein-HaloTag fusion as a means to normalize the fluorescence from a HaloTag ligand over time helps to follow the time-resolved behavior but also adds a source of influence on the apparent behavior that is not readily visible when only the normalized data are presented. In the discussion the authors mention that this normalization might be imperfect but only discuss one side of the imperfection: the overall influence of newly synthesized mTurq on total mTurq (which is indeed most likely not very high). What is missing from my point of view is the discussion of the influence of mTurq fluorescence decline over time as e.g. visible in Fig. 4B. Is this bleaching? What is the order of magnitude of this effect on the apparent increase in relative ligand fluorescence? At least for one of the figures the non-normalized data of all fluorescent partners could be presented alongside the normalized ones.
- The potential effect of residual CPXH labeling after washout on the onset of ligand labeling of newly synthesized protein should be discussed: Is there any detectable lag phase? Does the shape of the curve in the early time points give any hints that an ongoing labeling after washing is either negligible or should be considered? 
- Regarding the Oregon Green labeling: it is not clear to me whether the sentence ‘Interestingly, we noted that labeling with Oregon Green ligand was associated with strong non-specific labeling that washed out relatively slowly’ refers to transfected cells or all cells (I guess all). The visible variation of Oregon Green fluorescence in the images in 6B should be discussed. It could also be worth addressing how short labeling with different ligands and CPXH chase might help to determine how ‘reliable’ a fluorescent ligand can be used for time-resolved studies.

Related to Figure 7:

- Just out of curiosity and not necessary for description of the method at all: since often protein synthesis inhibitors are used at much shorter timescales – did you check how complete is the protein synthesis block judged by this method and under the conditions given after up to 1 hr?

Is the rationale for developing the new method (or application) clearly explained?

Yes

Is the description of the method technically sound?

Yes

Are the conclusions about the method and its performance adequately supported by the findings presented in the article?

Yes

If any results are presented, are all the source data underlying the results available to ensure full reproducibility?

Yes

Are sufficient details provided to allow replication of the method development and its use by others?

Yes

Reviewer Expertise:

neuronal cell biology, protein synthesis

I confirm that I have read this submission and believe that I have an appropriate level of expertise to confirm that it is of an acceptable scientific standard.

# Response

**Published:** 2020-06-02

**Authors:** Noam Ziv

**Affiliations:** Department of Anatomy and Cell Biology, Technion, Israel

We thank the reviewer for the supportive and constructive review.

 Responses below are interleaved with the original comments :

 Related to Figure 2:

- As important for the method in the text regarding the quantification in D the points of ‘complete’ blocking are discussed. It nonetheless would be helpful to also have a sentence on the data without and with low concentration of CPXH (e.g. high variability between time points in the 0µM).

Indeed. We now address these observations in the legend of Figure 2.

  

- Add a line how similar this is for neurons (was the concentration used in the following experiments based on the HEK data or determined independently).

The concentrations were based on measurements made in HEK cells. This is now stated in the main text.

  

- Toxicity of new compounds and also influence on physiology is an important issue (especially in neurons). The authors have addressed this by not only live/dead assays in HEK cells but also generated data by long-term CPXH incubation on neuronal network activity. This important dataset should be present in a main figure.

Data on toxicity, including MEA recordings, were moved to the main text (Figure 2E, F)

 Related to Figure 4-6/discussion:

- The use of a fluorescent protein-HaloTag fusion as a means to normalize the fluorescence from a HaloTag ligand over time helps to follow the time-resolved behavior but also adds a source of influence on the apparent behavior that is not readily visible when only the normalized data are presented. In the discussion the authors mention that this normalization might be imperfect but only discuss one side of the imperfection: the overall influence of newly synthesized mTurq on total mTurq (which is indeed most likely not very high). What is missing from my point of view is the discussion of the influence of mTurq fluorescence decline over time as e.g. visible in Fig. 4B. Is this bleaching? What is the order of magnitude of this effect on the apparent increase in relative ligand fluorescence? At least for one of the figures the non-normalized data of all fluorescent partners could be presented alongside the normalized ones.

This is an excellent point. These data were analyzed for all neurons and three examples are now shown in Figure 4D. We found that indeed, in some (about half of the neurons), some decay of mTurq2 fluorescence was observed (13% after 10 hours on average, up to 37% in the most extreme case). These observations and their implications are now detailed and discussed in the Results and Discussion.

- The potential effect of residual CPXH labeling after washout on the onset of ligand labeling of newly synthesized protein should be discussed: Is there any detectable lag phase? Does the shape of the curve in the early time points give any hints that an ongoing labeling after washing is either negligible or should be considered? 

This too is a good point. At the time scales and imaging frequency of the experiments described here, lag phases on the order of minutes would be undetectable. We found no overt evidence for lag phases on longer time scales, but until rigorous measurements of CPXH efflux rates are carried out, this confound should be brought into account. This is now mentioned explicitly in the Discussion.

  

- Regarding the Oregon Green labeling: it is not clear to me whether the sentence ‘Interestingly, we noted that labeling with Oregon Green ligand was associated with strong non-specific labeling that washed out relatively slowly’ refers to transfected cells or all cells (I guess all). The visible variation of Oregon Green fluorescence in the images in 6B should be discussed. It could also be worth addressing how short labeling with different ligands and CPXH chase might help to determine how ‘reliable’ a fluorescent ligand can be used for time-resolved studies.

This matter is now explained in much more detail in the main text.

 Related to Figure 7:

- Just out of curiosity and not necessary for description of the method at all: since often protein synthesis inhibitors are used at much shorter timescales – did you check how complete is the protein synthesis block judged by this method and under the conditions given after up to 1 hr?

No we did not; however, to do so one would need to use a protein that has a much higher turnover rate than mTurq2-HaloTag seems to have (as evidenced from Figures 4 to 6).

# Peer review: Reviewer response for version 1

**DOI:** 10.5256/f1000research.25710.r62839

**Published:** 2020-05-19

**Authors:** Timothy Ryan

**Affiliations:** 1 Department of Biochemistry, Weill Cornell Medical College, New York, NY, 10021, USA

The authors present a useful and insightful study on an approach that leverages genetically encoded Halo-tags fused to a fluorescent protein to show how new protein synthesis can be readily detected using a novel colorless blocking agent. The experiments are clear, analyzed in quantitative detail and the results appear very robust. I have the following suggestions which I think would improve this manuscript:

1. There is only a side mention of how the data of cell # 2 in Figure 6D should be interpreted. A cell was labeled with OG, chased with CPHX and the pulsed with JF646. Why would the OG fluorescence go down? Does this reflect some reversal of OG binding or purely background washout?
2. Related to this point the ability to use this scheme to get estimates of new protein synthesis does depend on the irreversibility of dyes binding to Halo. It seems this point could have been fleshed out a little more by asking how stable fluorescence in a fluorescent label followed by CPHX chase in more than just the anecdote in Fig 6.
3. It is probably worth discussing the fact that the measurements depend to a certain extent on the relative maturation times of the mTurq versus Halo proteins. I imagine is it not limiting on long time scales but could become so on shorter ones.

Is the rationale for developing the new method (or application) clearly explained?

Yes

Is the description of the method technically sound?

Yes

Are the conclusions about the method and its performance adequately supported by the findings presented in the article?

Yes

If any results are presented, are all the source data underlying the results available to ensure full reproducibility?

Yes

Are sufficient details provided to allow replication of the method development and its use by others?

Yes

Reviewer Expertise:

neuronal cell biology & physiology; development of quantitative optical methods

I confirm that I have read this submission and believe that I have an appropriate level of expertise to confirm that it is of an acceptable scientific standard.

# Response

**Published:** 2020-06-02

**Authors:** Noam Ziv

**Affiliations:** Department of Anatomy and Cell Biology, Technion, Israel

Thank you for the supportive and constructive review.

 Responses follow, in the same order as the commentary:

 1) This decay reflects slow OG ligand washout as determined by measuring OG fluorescence decay in somata and thick dendrites of neighboring, mTurq2 negative cells. The potentially slow efflux of this ligand is also documented by Promega ( Technical Manual, HaloTag® Technology: Focus on Imaging, page 35). This is now explained in the Results.

 2) We did not test the binding reversibility of any of the ligands, and this is indeed a potential confound in long experiments. We note that a) the literature indicates that HaloTag ligand binding is effectively irreversible and that b) CPXH is essentially the canonical HaloTag ligand lacking the reactive amine used for tagging it with fluorescent groups. Given the magnitude of the slow OG effect (comment #1), this is unlikely to be a major explanation for the observation in Fig. 6.  Nevertheless, we mention this potential confound in the Discussion.

 3) This is indeed a good point. We now mention it in the Discussion.
